# A Novel Multi-Resonator Polygonal Honeycomb Origami Metamaterial for Wave Transmission and Impact Mitigation

**DOI:** 10.3390/ma19153232

**Published:** 2026-07-29

**Authors:** Boyi Wei, Tengjiao Jiang, Chenyi Shen, Lingkai Wei, Dongliang Xiao

**Affiliations:** 1 National Engineering Research Center of Biomaterials, Nanjing Forestry University, Nanjing 210037, Chinajiangtengjiao@njfu.edu.cn (T.J.);; 2 School of Civil Engineering, Guangzhou City University of Technology, Guangzhou 510800, China

**Keywords:** origami metamaterials, local resonance, bandgap, mass-spring model, impact mitigation

## Abstract

Origami structures are recognized for their exceptional deformability and programmability, serving as a promising platform for designing mechanical metamaterials. In this paper, a local-resonant polygonal honeycomb origami metamaterial (LR-OHS) is proposed to achieve low-frequency wave attenuation and impact mitigation. The bandgap (BG), transmission spectrum, and mode analysis are investigated in detail through numerical calculations and experimental validation. It is demonstrated that two complete BGs in the low-frequency range are found, and the underlying generation mechanism of these BGs is elucidated theoretically by establishing a mass-spring model. Subsequent research discusses the influence of three significant parameters on the two complete BGs within the region of interest, as well as the broadening of the low-frequency BGs through the merger of two narrow BGs induced by an increasing resonator radius. Furthermore, the impact resistance performance of LR-OHS is evaluated under impact pulses, demonstrating a 43.43% reduction in the peak reaction force compared to its non-resonator origami honeycomb metamaterial. Additionally, parametric analysis of the number of resonators identified an optimal configuration of eight resonators per unit cell, ensuring high performance while satisfying lightweight engineering requirements. This work establishes a design framework for origami-based metamaterials, offering a viable path toward high-performance structures for wave attenuation and impact mitigation.

## 1. Introduction

Origami, while an ancient art form, is increasingly recognized in engineering and scientific research for the unique geometric characteristics of its structures. It exhibits considerable folding deformation and versatile geometric design capabilities. These characteristics provide origami structures with exceptional design freedom and anomalous properties, such as negative Poisson’s ratio [1,2,3,4], negative stiffness [5,6], and multistability [7,8,9,10,11]. Due to these excellent functionalities [12,13], origami metamaterials have promising application potential in various fields, including biomedical devices [14], robotics [15,16], battery manufacturing [17], and aerospace [18].

The study of vibrations is important from the point of view of the analysis of materials, structures, continuous systems with constraints, and controlled systems or oscillators. The dynamic behaviour of metamaterials could also be enhanced by adjusting the geometric parameters of the structure. Auxetic metamaterials and non-auxetic structures show different static and dynamic properties [19]. The dynamic properties of the analyzed structures were examined by the authors to determine the frequency ranges of dynamic loads for which the values of mechanical impedance and transmissibility are appropriate. Specifically, the analysis of mechanical impedance provides deep insight into the load-bearing capacity of structures under dynamic excitation [20].

Owing to their exceptional reconfigurability, programmability, and deformability, origami structures serve as an ideal platform for designing mechanical metamaterials. Meanwhile, the periodic feature of origami structures reveal significant potential for manipulating elastic waves, as evidenced by their application in wave mode conversion [21], bandgaps [22,23,24], and waveguides [25,26,27,28]. In general, there are two mechanisms for generating bandgaps: Bragg scattering and local resonance. The former is caused by multiple scattering and interference effects. Gao et al. [29] developed an origami-inspired periodic structure in which low-frequency bandgaps were successfully opened by utilizing Bragg scattering through geometric parameter optimization. Ye et al. [30] proposed a graded supercell structure composed of units with gradually varying structural parameters. This design merges multiple bandgaps by breaking symmetry, thereby forming an ultra-wide Bragg scattering bandgap. Jiang et al. [31] designed a multi-scale origami metamaterial. They used a semi-analytical method to study wave propagation and demonstrated tunable Bragg bandgaps via parametric analysis. However, a limitation of the Bragg scattering mechanism is that the bandgap frequency is inversely proportional to the lattice constant, which necessitates the use of larger structures to achieve low-frequency bandgaps. The latter is the local resonance mechanism. It was first derived by Liu et al. [32] that low-frequency bandgaps can be realized through the local resonance mechanism of the unit cells, thereby providing a new approach for the low-frequency vibration reduction in small-size periodic structures [33,34,35]. Wang et al. [36] proposed a piezoelectric metamaterial with local resonators, and their analysis of the coupling effect via piezoelectric shunting revealed a significant improvement in low-frequency bandgaps. Sugino et al. [37] investigated a metamaterial with a finite number of resonators using modal analysis. They examined the influence of the resonator number on the bandgap, leading to the identification of an optimal resonator count. On this basis, the integration of local resonance principles with origami structures has currently yielded numerous groundbreaking advances. Yu et al. [38] developed a one-dimensional nonlinear resonant origami metamaterial. Through theory and experiments, they showed that excitation amplitude tunes bandgaps via nonlinear coupling stiffness. Through numerical simulations, they showed that the bandgaps can be effectively tuned by parametric analysis.

Despite the extensive research on low-frequency and wide bandgaps, simultaneously considering low-frequency wave attenuation and impact resistance is still a great challenge for metamaterial design. Furthermore, with the continuous development of the local resonance mechanism, unique advantages and profound significance have been demonstrated by its research in the field of impact resistance. Kim et al. [39] demonstrated a locally resonant woodpile metamaterial, investigated the coupling mechanism between nonlinear stress waves and local bending vibration modes of rod elements, and achieved a reduction in shock wave transmission. Li et al. [40] presented a multi-resonator metamaterial and studied the effects of resonator number, stiffness, and mass. Their theoretical and numerical analysis confirmed that this design achieves wider bandgaps and superior impact resistance compared to single-resonator and dual-resonator. Zhou et al. [41] developed a dual locally resonant metamaterial, investigated the dynamic load attenuation capacity of the multi-resonator system under varying impact durations, and demonstrated that the synergy between local and diatomic resonance significantly reduces impact force transmissibility. Zheng et al. [42] designed a honeycomb metamaterial with circular mass-beam resonators. Numerical and analytical studies showed its high impact resistance, achieved by converting impact kinetic energy into the kinetic energy of the resonator core. Li et al. [43] proposed a single-resonator metamaterial with negative mass. Their numerical study on spring stiffness and unit cell count revealed that a critical number of units and variable stiffness design can more effectively attenuate shock waves.

As shown from the above research, although there have been many studies on local resonance in terms of impact mitigation, research on the integration of origami structures is relatively limited. Zhang et al. [44] developed origami-based bistable metastructures employing bistable origami configurations as resonant unit cells, investigated their tunable vibration isolation performance, and achieved active control of the vibration isolation frequency range. Li et al. [45] developed a hybrid graded origami metamaterial with multiple resonators. Their numerical simulations demonstrated that asymmetric, graded configurations significantly enhance wave energy dissipation through multi-local resonance. Han et al. [46] developed an arch-honeycomb metamaterial incorporating split-ring resonators, investigated the effects of the structure on wave attenuation and impact mitigation, and demonstrated its integrated multifunctional capabilities. Based on the above research content, the incorporation of local resonance mechanisms and origami-inspired structures not only expands the versatility and diversity in metamaterial topological design, but also significantly enhances their functional performance, thereby broadening potential applications.

In recent years, to expand the applications of origami structures and overcome the inferior deformation capacity of conventional honeycombs, researchers have combined origami structures with traditional honeycombs to meet increasingly strict mechanical performance requirements. However, research on origami honeycombs has focused predominantly on their mechanical properties, while the wave characteristics and impact mitigation performance under local resonance mechanisms have not been extensively explored. Therefore, in this paper, a local-resonant polygonal honeycomb origami metamaterial is proposed through an evolutionary design process. In Section 1, the structural design is presented and a characteristic mode analysis of the LR-OHS is performed. In Section 2, the wave propagation and attenuation characteristics are analyzed using both experimental verification and numerical simulation methods. The number and width of the bandgaps are examined through a parametric study. Moreover, a mass-spring system is introduced to theoretically elucidate the mechanism of bandgap generation. In Section 3, in addition to the conventional parameter analysis on the impact resistance of the structure, this paper studies the influence of the number of oscillators on impact mitigation properties in greater depth.

Several locally resonant origami and honeycomb metamaterials have recently been proposed for wave attenuation and impact mitigation. Compared to these existing designs, the proposed LR-OHS uniquely integrates two complete low-frequency bandgaps and a 43.43% reduction in peak impact reaction force within a single origami-honeycomb architecture, while also enabling bandgap merging through parameter modulation. This combination of multi-bandgap attenuation and significant impact mitigation has not been simultaneously achieved in prior works, highlighting the novel contribution of this study.

## 2. Design and Method

### 2.1. Structure Design and Geometric Parameters

To address practical engineering requirements, a local-resonant polygonal honeycomb origami metamaterial is proposed by this study, in which Miura-ori patterning is incorporated into a conventional honeycomb design. A tunable low-frequency bandgap is achieved by this configuration, and excellent impact resistance is exhibited. Furthermore, the coalescence of multiple narrow bandgaps into a broad bandgap is enabled by parameter modulation, and low sensitivity to specific geometric parameters is demonstrated by this broad bandgap.

**Figure 1:**
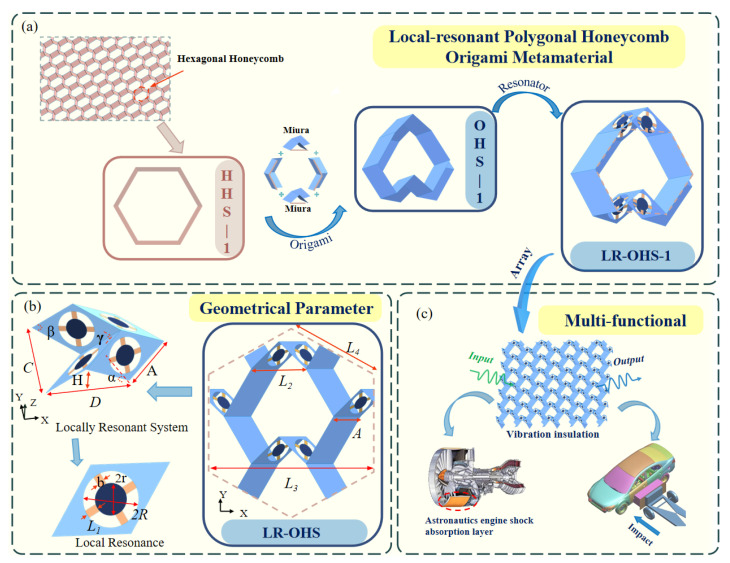
Design concept of a local-resonant polygonal honeycomb origami metamaterial(LR-OHS): (**a**) Design concept of the proposed metamaterials; (**b**) Schematic of the LR-OHS unit cell with significant geometric parameters labeled; (**c**) Potential applications of LR-OHS.

As shown in Figure 1a, Miura-ori patterns are used to replace the top and bottom edges of the conventional polygonal honeycomb structure (HHS-1). The origami honeycomb structure (OHS-1) is then formed by connecting these patterns along the remaining four edges through origami folding. Subsequently, the locally resonant origami honeycomb structure (LR-OHS-1) is created by the introduction of local resonators onto each facet of the Miura-ori. The substrate of the origami structure is fabricated from epoxy. The resonant masses within the Miura-ori cells are made of lead. Each mass is connected to the substrate via four narrow beams made of rubber, which function as equivalent springs. Owing to the periodic nature of the structure, the fundamental unit cell for the array is constituted by the polygonal LR-OHS unit shown in Figure 1b, and the same principle is applied to the other two structures. The lattice constant of the unit cell, denoted as $L_{3}$, is governed by the geometric parameter *D* of the Miura-ori pattern. The lattice constants in the x- and y-directions are defined by parameters *C* and *D*, respectively, alongside the dependent variable *H*. Here, the sector angle of the Miura-ori is represented by β, and the folding angle is denoted by α. The remaining geometric details and material properties are summarized in Table 1 and Table 2, respectively.

(1) $$\begin{array}{c} L_{3}=3L_{2},L_{2}=D,D=2A{\left(1-\sin^{2}\alpha \sin^{2}\beta \right)}^{\frac{1}{2}} \end{array}$$

(2) $$\begin{array}{c} H=A\sin\alpha \sin\beta ,C=2A\frac{\cos\alpha \tan\beta }{{\left(1+\cos^{2}\alpha \tan^{2}\beta \right)}^{\frac{1}{2}}} \end{array}$$

(3) $$\begin{array}{c} \sin\left(\frac{\gamma }{2}\right)=\frac{\cos\alpha }{\cos\beta {\left(1+\cos^{2}\alpha \tan^{2}\beta \right)}^{\frac{1}{2}}} \end{array}$$

### 2.2. Calculation Method

#### 2.2.1. Band Structure

All the numerical simulations of the bandgap in this paper were carried out using COMSOL Multiphysics 6.0. The solid mechanics module is used for the band structure calculation, and Bloch–Floquet periodic boundary conditions were applied at the boundaries of the unit cell. For band structure calculations in the unit cell, the Solid Mechanics study is used with Bloch–Floquet periodic boundary conditions applied to all boundaries of the unit cell. The wave vector *k* was swept along the O→A→B→C→D→E→O path of the irreducible Brillouin zone. For the transmission spectrum of the 10 cell finite array, a frequency domain analysis is performed, sweeping the frequency from 10 Hz to 5 kHz. A free triangle mesh with quadratic serendipity shape functions is used for all models. The deformations in our studied bandgap and impact regimes are small, and the rubber springs operate within their linear viscoelastic range. Therefore, all materials are treated as linear elastic.

**Figure 2:**
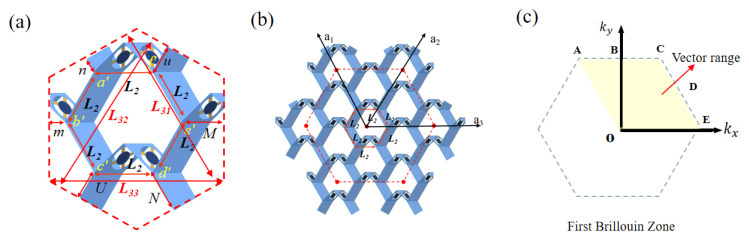
(**a**) LR-OHS unit cell. (**b**) Array form and basis vectors of a unit cell. (**c**) The first Brillouin zone (highlighted in yellow).

As shown in Figure 2a, at the initial stage of structural design, the lattice length *D* of the Miura-ori unit always equals the side length $L_{2}$ of the regular hexagon, i.e., $L_{a^{\prime }b^{\prime }}=L_{b^{\prime }c^{\prime }}=L_{c^{\prime }d^{\prime }}=L_{d^{\prime }e^{\prime }}=L_{e^{\prime }f^{\prime }}=L_{f^{\prime }a^{\prime }}=L_{2}$, ensuring that the hexagon $a^{\prime }b^{\prime }c^{\prime }d^{\prime }e^{\prime }f^{\prime }$ remains a regular hexagon. Moreover, $m+M=n+N=u+U=L_{2}$, resulting in $L_{31}=L_{32}=L_{33}=L_{3}=3L_{2}$, making the LR-OHS lattice a regular hexagonal structure with a lattice length of $L_{3}$. Figure 2b presents the unit cell configuration and the basis vectors. The first Brillouin zone is defined in Figure 2c. The wave vector is parametrically scanned along the boundary of the irreducible Brillouin zone, which is highlighted in yellow in Figure 2c. The wave vector path within this zone is denoted as O→A→B→C→D→E→O. To investigate the propagation and dynamic characteristics of elastic waves in this honeycomb metamaterial, the elastic wave equation is employed. For a heterogeneous, isotropic, linearly elastic medium, the time harmonic governing equation for the displacement field u(x,t) can be written in index notation or vector form. In vector form, the equation is [47]

(4) $$\nabla \left[\lambda \left(s\right)+2\mu \left(s\right)\right]\nabla \cdot u-\nabla \times \left[\mu \left(s\right)\left(\nabla \times u\right)\right]=\rho \left(s\right)\frac{{𝜕}^{2}u}{𝜕t^{2}}$$

where λ and μ are the first and second Lamé constants of the material, respectively, which are piecewise constant within each material domain but vary spatially across different materials in the heterogeneous unit cell. ρ is the material density, and u is the displacement vector, which is a function of the position vector s=(x,y). According to the Bloch theorem, the solution for the displacement field in a periodic structure can be expressed in the form:

(5) $$u\left(x,t\right)=e^{i(kx-wt)}U_{k}\left(x\right)$$

where $U_{k}\left(x\right)$ is a periodic function with the same periodicity as the structure, and *k* is the Bloch wave vector, ω is the angular frequency, which satisfies

(6) $$U_{k}\left(x+R_{l}\right)=U_{k}\left(x\right)$$

Here, $R_{l}$ represents the lattice translation vector. Generalized eigenvalue problem is generally expressed as

(7) $$K_{i}U_{i}=\omega^{2}M_{i}U_{i}+F_{i}$$

In the eigenvalue equation presented above, $K_{i}$ represents the stiffness matrix; $M_{i}$ denotes the global mass matrix; $U_{i}$ and $F_{i}$ are the displacement amplitude vector and the force vector at the generalized nodes, respectively. The band structure is obtained through numerical simulation by scanning the wave vector along O→A→B→C→D→E→O of the irreducible Brillouin zone, as shown in Figure 2, for a unit cell with defined dimensions and material parameters. While the introduction of the origami pattern and resonators reduces the symmetry of the unit cell from that of an ideal hexagonal lattice, the underlying Bravais lattice remains hexagonal. The chosen path O→A→B→C→D→E→O traverses the perimeter of the first Brillouin zone and is sufficient for identifying the complete bandgaps.

#### 2.2.2. Transmission Spectrum and Equivalent Mass Density

**Figure 3:**
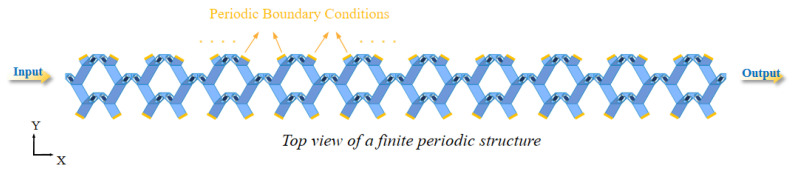
Finite structure model used for calculating transmission spectra.

To validate the bandgap characteristics of LR-OHS, the transmission loss of elastic waves propagating along the x-direction in a finite structure comprising 10 unit cells is numerically calculated, as shown in Figure 3. A displacement excitation is applied to the left boundary of the structure along the positive x-direction, and the corresponding displacement response is recorded at the right boundary. Simultaneously, periodic boundary conditions are applied to the top and bottom boundaries in the y-direction to simulate an infinite periodic extension. The transmissibility is then calculated using the following equation:

(8) $$T=20\log_{10}\left(\left|\frac{U_{Out}}{U_{In}}\right|\right)$$

Here, *U* denotes the root mean square (RMS) of the total displacement, which is computed from the complex displacement field *u* over the output/input boundary as

(9) $$U=\sqrt{\frac{1}{A}\int_{A}{∥u∥}^{2}dA}$$

where *A* is the area of the respective boundary. The subscripts ‘Out’ and ‘In’ correspond to the output and input ends of the model shown in Figure 3, respectively.

To more accurately predict wave propagation characteristics in the proposed metamaterial, effective material parameters are derived using effective medium theory. Based on the long-wavelength assumption and principles of continuum mechanics, the unit cell of this metamaterial can be treated as a macroscopically homogeneous effective medium. The effective dynamic mass density governing elastic wave propagation in the LR-OHS unit cell is derived from its global displacement field:

(10) $$\rho_{eff}=-\frac{F_{0}}{w^{2}V_{AVE}U_{0}}$$

In the above equation, $V_{AVE}$ represents the volume of the unit cell, $F_{0}$ is the net reaction force in the direction of wave propagation summed over the boundary where the displacement is prescribed, and $U_{0}$ is the global displacement field. The global displacement $U_{0}$ is computed as the volume-averaged displacement in the direction of wave propagation over the entire unit cell, i.e., $U_{0}=\frac{1}{V_{AVE}}\int_{V}u_{x}dV$.

#### 2.2.3. Mass-Spring Systems

**Figure 4:**
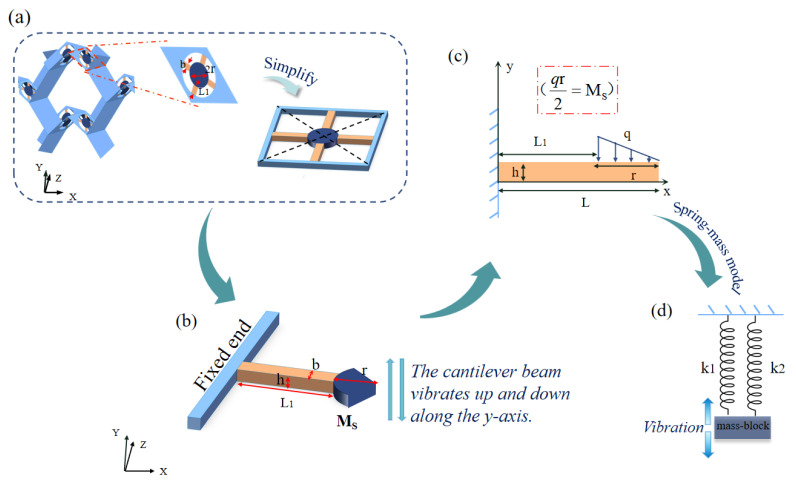
Simplification process of the spring-mass system: (**a**) Schematic of the simplified resonant system; (**b**) Fundamental unit of the resonant system; (**c**) Simplified cantilever beam model; (**d**) Equivalent spring-mass model with two parallel springs.

In locally resonant metamaterials, the center frequency of the low-frequency bandgap depends on the characteristic frequency of the local resonant system. Therefore, a simplified mass-spring system is constructed to model the LR-OHS, as shown in Figure 4b. The characteristic frequency of this system is derived analytically to validate the accuracy and scientific basis of the bandgap results. It is revealed by the derivation of the formula for the characteristic frequency that it is highly sensitive to the resonator radius and the rubber beam width. A detailed parametric study of these influences is presented in the following section. The internal resonant unit of the LR-OHS can be simplified into the mass-spring system depicted in Figure 4b, based on the detailed model in Figure 4a. The dark blue sector in Figure 4c is simplified to a triangularly distributed load that increases linearly toward the fixed end. The equivalence is established by $\mathrm{qr}/2=M_{S}$, where $M_{S}$ is the actual mass of the sector, *q* represents a triangularly distributed load, given by $M_{S}=0.25\pi r^{2}$. Here, *r* represents the radius of the sector. Owing to the complexity of the load and its non-uniform distribution over the entire length *L*, the equivalent spring stiffness is defined in two segments: $K_{1}$ for x∈[0,L−r] and $K_{2}$ for x∈[L−r,L]. These two springs, $K_{1}$ and $K_{2}$, are connected in parallel and jointly attached to the mass block $M_{S}$.

(I) For x∈[0,L−r].

According to Hooke’s law,

(11) $$\frac{qr}{2}=K_{1}\zeta_{1}$$

Taking into account the segment from the fixed end to the point x=L−r as a rigid body, the bending moment at a distance *x* from the fixed end is the following:

(12) $$M\left(x\right)=\frac{qr}{2}\left(L-r-x\right)$$

The Euler–Bernoulli beam equation is employed, $EI\ddot{y}=M\left(x\right)$ (where $\ddot{y}$ denotes the curvature). The slope, $\dot{y}$ is obtained by integrating this equation once with respect to *x*, and the deflection, *y*, is obtained by a second integration. The boundary conditions at the fixed end x=0, where both the deflection and slope are zero, are applied to determine the constants of integration. The expression for M(x) from Equation (11) is substituted into the Euler–Bernoulli equation, and the result is obtained:

(13) $$EIy=\left(\frac{qr}{4}\right)\left(L-r\right)x^{2}-\left(\frac{qr}{12}\right)x^{3}$$

The deflection at x=L−r is denoted as $\zeta_{1}$ substituting Equation (13) and simplifying yields

(14) $$\zeta_{1}=\frac{qr{\left(L-r\right)}^{3}}{6EI}$$

Subsequently, the equivalent spring stiffness $K_{1}$ for the first segment is derived from Hooke’s law:

(15) $$K_{1}=\frac{3EI}{{\left(L-r\right)}^{3}}$$

(II) For x∈[L−r,L].

A procedure analogous to that for the first segment is applied. Based on Hooke’s law, the following relation is obtained:

(16) $$\frac{qr}{2}=K_{2}\zeta_{2}$$

Due to the non-uniform mass distribution in the fan-shaped segment—where the mass decreases with increasing distance from the fixed end—the region to the right of x=L−r is modeled as being subjected to a tapered triangular distributed load. Consequently, the bending moment at a distance *x* from the fixed end is given by

(17) $$M\left(x\right)=\frac{q{\left(L-x\right)}^{3}}{6r}$$

The Euler–Bernoulli beam equation is integrated once with respect to *x* to obtain the slope, and a second time to obtain the deflection. In contrast to the first segment, the boundary conditions at x=L−r are nonzero constants. These values, derived from the solution for the first segment, as following:

(18) $$y=\frac{qr{\left(L-r\right)}^{3}}{6EI};\frac{dy}{dx}=\frac{qr{\left(L-r\right)}^{2}}{4EI}$$

The deflection at the free end (x=L) is denoted as $\zeta_{2}$ and is given by

(19) $$\zeta_{2}=\frac{C_{11}L+C_{22}}{EI}$$

where $C_{11}=\left(6qr{\left(L-r\right)}^{2}+qr^{3}\right)/24$; $C_{22}=-\left(10qr{\left(L-r\right)}^{3}+qr^{4}+5qr^{3}\left(L-r\right)\right)/120$. The equivalent spring stiffness $K_{2}$ for the segment with the triangular distributed load is then derived from Hooke’s law:

(20) $$K_{2}=\frac{2qrEI}{2(C_{11}L+C_{22})}$$

The two springs are in parallel; thus, the total equivalent spring stiffness *K* is the sum of the individual stiffnesses: $K=K_{1}+K_{2}$. The characteristic frequency (*f*) of the structure is subsequently determined from its characteristic frequency formula:

(21) $$f=\frac{\sqrt{\left(K/M_{S}\right)}}{2\Pi }$$

## 3. Results and Discussion

### 3.1. Band Structure and Wave Transmission

#### 3.1.1. Bandgap Evolution

**Figure 5:**
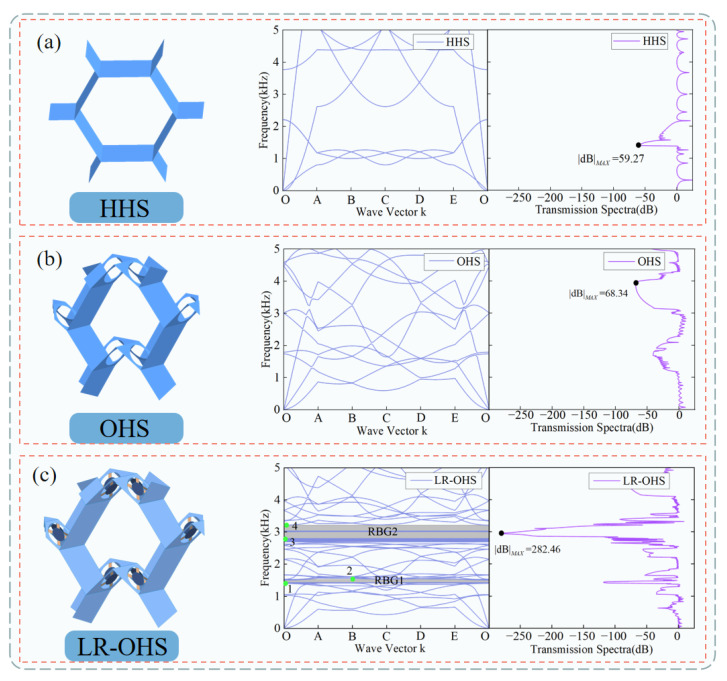
Comparative evolution of bandgaps: (**a**) Band structure and transmission spectrum of the HHS; (**b**) Band structure and transmission spectrum of the OHS; (**c**) Band structure and transmission spectrum of the LR-OHS, and the gray shaded region indicates the complete bandgap generated by this structure.

The influence of the origami structure and resonators on the bandgaps is shown in Figure 5. The band structure and transmission spectrum of the corresponding metamaterials are represented by the blue and purple curves, respectively. For the HHS (Figure 5a), neither directional bandgaps nor complete bandgaps are exhibited by the band structure. The maximum attenuation in the transmission spectrum is merely 59.27 dB. The introduction of the origami pattern to form the OHS (Figure 5b) results in only a marginal improvement. A single directional bandgap is opened in the 0–5 kHz range, with a maximum attenuation of 68.34 dB, which is only 15.3% higher than that of the HHS. In contrast, two distinct low-frequency bandgaps are opened by the LR-OHS, in which local resonators are incorporated. The first bandgap RBG1, with a bandwidth of 95.5 Hz, is spanned from 1.41 kHz to 1.51 kHz, and the second bandgap RBG2, with a bandwidth of 0.56 kHz, is spanned from 2.66 kHz to 3.21 kHz. Within these bandgap frequency ranges, the peak attenuation reaches 282.46 dB. This value is approximately 4.8 and 4.1 times greater than the transmission minima of the HHS and OHS, respectively. It is demonstrated that vibrational energy is effectively absorbed and localized by the introduced resonators, thereby resulting in the propagation of elastic waves within the structure being inhibited more effectively.

#### 3.1.2. Bandgap Mechanism

**Figure 6:**
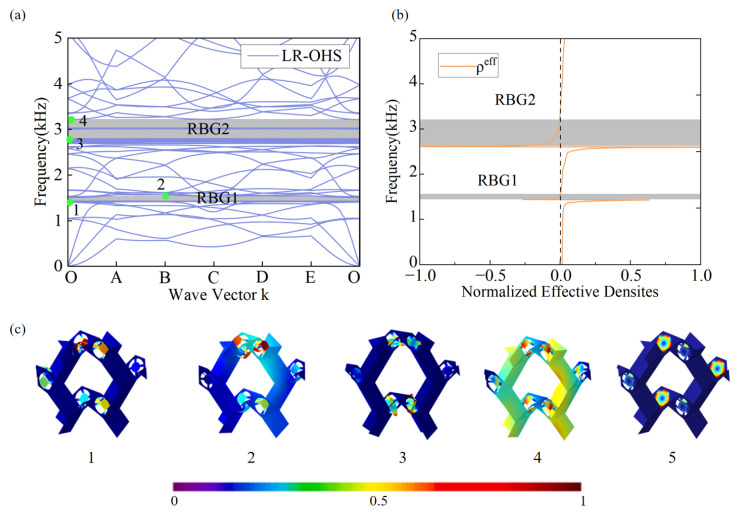
Bandgap mechanism of the LR-OHS: (**a**) Band structure of the LR-OHS. Points 1 and 2 mark the upper and lower boundaries of the RBG1, while points 3 and 4 correspond to those of the RBG2. (**b**) Equivalent mass density of the LR-OHS. (**c**) Displacement fields under different modes.

To elucidate the mechanism underlying the low-frequency bandgaps, the characteristic displacement modes at four significant frequencies corresponding to the upper and lower boundaries of the RBG1 and RBG2 in the LR-OHS are presented in Figure 6c. For modes 1 and 2 of the RBG1, significant out-of-plane translation is undergone by the resonators; that is, their displacement is perpendicular to the plane of the substrate facets. Critical justification for the simplified mass-spring system model developed in the subsequent theoretical analysis is provided by this observation. Mode 3 is characterized primarily by rotational vibration about the axis defined by the two symmetric rubber beams, whereas mode 4 is dominated by in-plane translation. It is revealed by a comparison of these four modes that resonator motion is out-of-plane in modes 1 and 2, and in-plane in modes 3 and 4. In all cases, the displacement of the substrate plates is relatively negligible, and it is confirmed that the vibration is highly localized within the resonant mass blocks. Within the bandgap frequency range, strong coupling with the local resonant units is experienced by incident elastic waves. Pronounced local resonance modes are excited by this coupling. Through these modes, the wave energy is channeled into the resonators. The energy is subsequently dissipated within these units, resulting in strong vibrational attenuation and the formation of the bandgap. Therefore, the introduction of a local resonance mechanism into the origami structure enables the opening of multiple low-frequency bandgaps. Through this mechanism, superior elastic wave attenuation performance is achieved. We found that mode 5 is located in the middle of RBG2. It can be seen from Figure 6c that 5 exhibits chiral modes. Although this mode is located in the middle of the bandgap, its range is extremely small and relatively smooth, and it hardly affects the total bandwidth and the subsequent parametric analysis.

Furthermore, the normalized effective mass density of the LR-OHS is plotted as the orange curve in Figure 6b. A remarkable coincidence is observed between the frequency range of its negative effective mass density and the bandgaps (1.41–1.51 kHz and 2.66–3.21 kHz) in the band structure (blue curve, Figure 6a), as well as with the primary transmission loss minimum in Figure 5c. This strong correlation further confirms that the bandgap in LR-OHS is caused by a strong local resonance effect. The bandgap formation arises from the local resonance mechanism, which creates a frequency region of negative effective mass density where wave propagation is prohibited. The subsequent attenuation amplitude within the bandgap is enhanced by the viscoelastic damping of the rubber springs, which dissipates the kinetic energy localized in the resonators.

#### 3.1.3. The Influence of Important Resonator Radius (r) Parameters on BGs

The bandgaps generated by local resonances introduced via oscillators are often highly sensitive to structural parameters. In contrast, complex and programmable deformation modes are inherently possessed by origami structures. The strong mechanical coupling between these two systems gives rise to novel properties. As a result, the emergent properties are far richer and more tunable than the mere sum of their individual parts. Parametric analysis serves as an effective approach to understand and harness this complexity. Therefore, building upon the theoretical foundation established in the preceding section, a deeper interpretation of how parametric studies influence the structural bandgaps will be provided.

**Figure 7:**
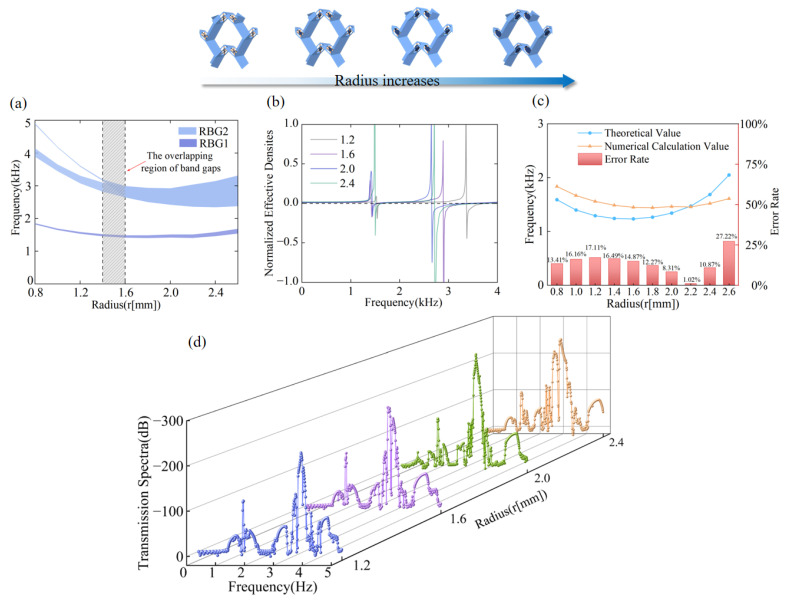
(**a**) Influence of oscillator r on the bandgap; (**b**) Influence of r on the effective mass density; (**c**) The law governing the influence of r on the error between theoretical and numerical simulation results for the RBG1; (**d**) Influence of r on the transmission spectrum.

Firstly, the resonator r is a critical geometric parameter and a primary source of error in the subsequent theoretical analysis. The unit cell with an increasing resonator r is schematically illustrated by the top panel of Figure 7. The influence of the r on the bandgap frequency ranges is shown by Figure 7. It is evident that the start and cut-off frequencies, center frequency, and width of both the RBG1 and RBG2 initially decrease and then increase with increasing resonator diameter. It is indicated by Equation (19) that the characteristic frequency *f* of the system is determined by the equivalent spring stiffness *K* and the resonator mass *M*, following the relationship f=K/M. For a small r within a certain range, an increase in r leads to a greater resonator mass. Concurrently, the rubber length is decreased, which causes its equivalent stiffness to be increased. However, in this regime, the increase in mass *M* is the dominant factor, resulting in a decrease in the characteristic frequency. Beyond a certain r, the rubber length is significantly reduced, causing its equivalent stiffness to become the dominant factor. Consequently, the characteristic frequency is increased. The widening of the bandgaps, particularly the RBG2, can be attributed to the local resonance mechanism. According to this principle, the width of a locally resonant bandgap is proportional to the resonator mass. The observed broadening of the bandgaps with increasing r is explained by this. Furthermore, as shown in Figure 7b, the frequency range of the negative effective mass density is shifted toward higher frequencies as the r increases, a trend that is consistent with the variation in bandgap width. Moreover, as shown in Figure 7d, within a certain range, the propagation of elastic waves in the structure is effectively inhibited, regardless of the variation in resonator r.

Beyond the conventional parametric analysis above, it is revealed by data processing that the two narrow bandgaps are merged into a single broad bandgap RBG2 at an r between 1.4 mm and 1.6 mm in Figure 7a. This merging is attributed to the fact that at small r, the two resonant modes remain independent and their frequencies are well-separated. Consequently, two separate, narrow bandgaps are produced by them. However, as the r increases, the frequencies of both modes are shifted toward lower frequencies. Moreover, with increasing r, the modes become coupled, resulting in a loss of independence of their vibrational patterns. This coupling leads to a gradual overlap of the two bandgaps, eventually resulting in their fusion into a continuous broad bandgap. Furthermore, from the observation of Figure 7a,b, it is indicated that for the RBG1, only the width is gradually increased with the r, while its center frequency remains relatively stable. In contrast, significant shifts are exhibited by both the width and the center frequency of the RGB2 as the r increases. It is indicated by this that the first center frequency is the most stable and low sensitivity to changes in resonator mass is exhibited. Conversely, the second center frequency can be tuned precisely and over a wide range by varying the resonator r, and high sensitivity to this parameter is demonstrated. It is revealed by this phenomenon that the interaction between waves and the structure within the local resonance framework is multi-level and multi-scale. As shown in Figure 6c, the coupling of different modes with elastic waves occurs in distinct ways and with varying strengths. The understanding of “modal-selective coupling” can be deepened by investigating these differences, and a foundation for designing more complex functional devices can be provided. Furthermore, the flexibility of structural design is significantly enhanced and the functionality of devices is enriched by it. The way for achieving multi-frequency wave control is paved by this advancement, transitioning from single to multiple functionalities and from passive to actively tunable operation.

The error between the theoretically calculated characteristic frequency and the center frequency from numerical simulation is shown by Figure 7c. The numerically calculated bandgap center frequency and the theoretically derived characteristic frequency are represented by the orange and blue curves, respectively. As can be seen, the error is minimized (1%) at a = 2.2 mm, and increased as the r deviates from this value. This occurs because a perfectly uniform mass distribution is assumed by the simplified sector-shaped resonator model. As the r decreases, the actual mass becomes more concentrated. This concentration causes the actual triangular load distribution to be narrower than the theoretical distribution assumed in Figure 6c, resulting in an underestimation of the equivalent spring stiffness *K*. Consequently, the theoretical characteristic frequency is lower than the numerical simulation center frequency for r less than 2.2 mm. Conversely, for r larger than 2.2 mm, non-uniform mass distribution is exacerbated by the increasing sector size. This leads to an actual load distribution that is wider than that assumed by the theoretical model, causing an overestimation of the spring stiffness *K*. This overestimation causes the theoretical frequency to exceed the numerical simulation value. Therefore, at r of approximately 2.2 mm, the theoretical error induced by the non-uniform mass distribution of the sector-shaped resonator becomes negligible.

#### 3.1.4. The Influence of Important Resonator Width (b) Parameters on BGs

**Figure 8:**
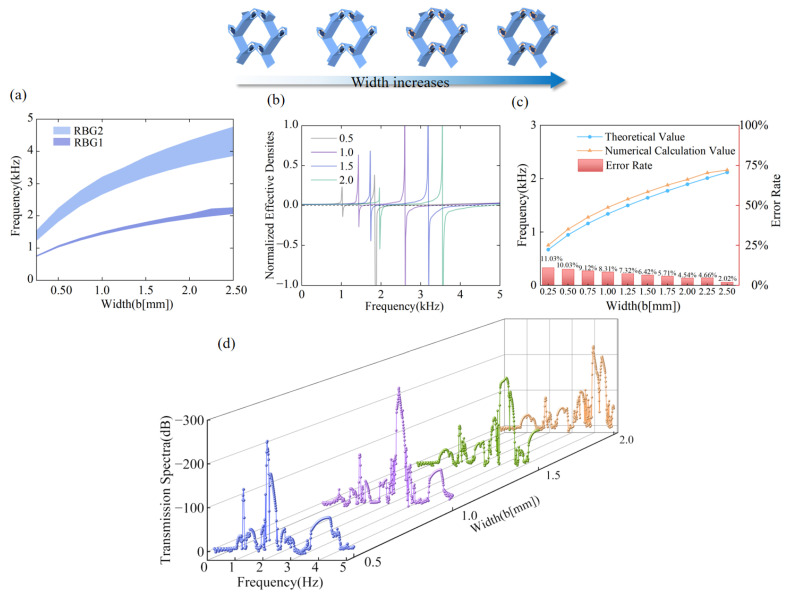
(**a**) Influence of b on the bandgap; (**b**) Influence of b on the effective mass density; (**c**) The law governing the influence of b on the error between theoretical and numerical simulation results for RBG1; (**d**) Influence of b on the transmission spectrum.

Furthermore, the b is another critical geometric parameter. The evolution of bandgaps with varying b is investigated in this section. The b is varied from 0.25 mm to 2.5 mm in increments of 0.25 mm. Schematic diagrams of the unit cell with progressively increasing b are presented in the top panel of Figure 8. As shown in Figure 8a, the center frequencies of both the RBG1 and RBG2 local resonance bandgaps shift toward higher frequencies with increasing b. This trend is corroborated by the corresponding shift in the negative effective mass density region in Figure 8b. The frequency shift occurs under a constant effective mass because the increase in b results in a greater equivalent stiffness. According to the characteristic frequency formula, this increase in stiffness leads to a higher local resonant bandgap center frequency. As shown in Figure 8d, within a certain range, the propagation of elastic waves in the structure is effectively inhibited regardless of the variation in b.

Based on the analysis in the previous section, the resonator r is identified as the primary source of error between theoretical and numerical simulation results. Figure 7c shows that the influence of non-uniform mass distribution in the sector-shaped resonator is minimized at an r of approximately 2.2 mm. Therefore, for the parametric analysis of b in this section, a fixed resonator r of 2 mm is selected. Figure 8c shows the error between the theoretical and numerical simulation values for a fixed resonator r of 2 mm and varying b. As before, the numerically calculated bandgap center frequency and the theoretically derived characteristic frequency are denoted by the orange and blue curves, respectively. The percentage error decreases gradually with increasing b, reaching a minimum of 2.02%. This trend can be explained as follows: at smaller b, the equivalent stiffness $K_{1}$ contributed by the rubber itself is relatively small when compared to the stiffness component $K_{2}$, which is derived from the triangular load distribution. Furthermore, although the absolute error may be similar, a decreasing b results in a lower numerically calculated center frequency. The denominator in the error percentage calculation is reduced by this, which is also a major reason for the source of error. Thus, as shown in Figure 8d, the theoretical and numerically calculated center frequency curves are in close agreement, nearly overlapping. The accuracy and scientific soundness of the numerical simulation results against the theoretical model are validated by this close agreement.

#### 3.1.5. The Influence of Important Dihedral Angle Parameters on BGs

**Figure 9:**
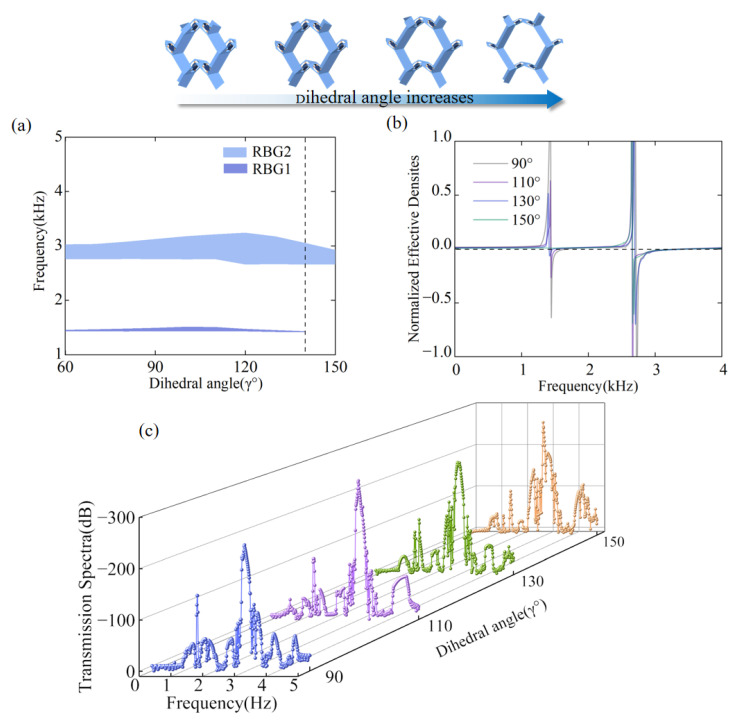
(**a**) Influence of the dihedral angle on the bandgap; (**b**) Influence of the dihedral angle on the effective mass density; (**c**) Influence of the dihedral angle on the transmission spectrum.

Similarly, the influence of the geometric parameter known as the Miura-ori dihedral angle is investigated in this section. How variations in this angle alter the unit cell shape is illustrated by the schematic at the top of Figure 9. The dihedral angle is examined over a range from 60° to 150° in increments of 10°. As shown in Figure 9a, the first complete bandgap vanishes when the dihedral angle exceeds 140°. Furthermore, a non-monotonic variation with increasing dihedral angle is exhibited by both the bandwidth and central frequency of the RBG1. For the RBG2, a trend of initial increase followed by a decrease as the dihedral angle grows is demonstrated by both the upper cut-off frequency and the central frequency. It is revealed by comparative analysis of Figure 9b,c that a negligible impact on the structural resonant frequencies is generated by the variation in the dihedral angle. Consistent with the findings in the previous two sections, the propagation of elastic waves is effectively impeded by the structure regardless of the change in this parameter.

#### 3.1.6. The Influence of Important Resonator Quantities Parameters on BGs

**Figure 10:**
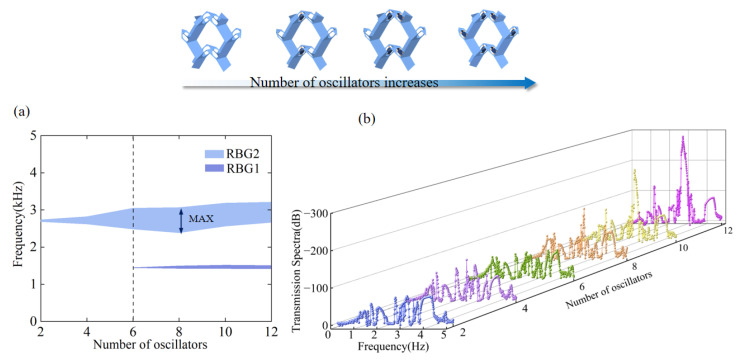
(**a**) Influence of the number of oscillators on the bandgap; (**b**) Influence of the number of oscillators on the transmission spectrum.

The effect of the number of oscillators on local resonance bandgaps is examined in this section. The unit cell with varying numbers of oscillators is depicted by the schematic at the top of Figure 10. The number of oscillators is varied from 2 to 12 in increments of two. As shown in Figure 10a, the RBG1 is initially opened when the number of oscillators reaches six. Concurrently, the bandwidth of RBG2 gradually expanded with a further increase in the number of oscillators. When the number of oscillators is eight, the maximum width of the RBG2 is reached. As shown in Figure 10b, a distinct attenuation peak appears at this count, signifying improved wave attenuation performance. However, the optimal attenuation is not achieved with eight oscillators. The most effective wave attenuation is achieved when the number of oscillators is increased to twelve. It is well-established that bandgaps in locally resonant metamaterials originate from the resonance of local oscillators. However, a bandgap is not necessarily generated by all resonant modes. As shown in Figure 10a, no bandgap is formed with a small number of oscillators due to insufficient coupling and a lack of cooperative interaction. The bandgap emerges only when a sufficient number of oscillators are arranged periodically.

### 3.2. Experimental Verification

**Figure 11:**
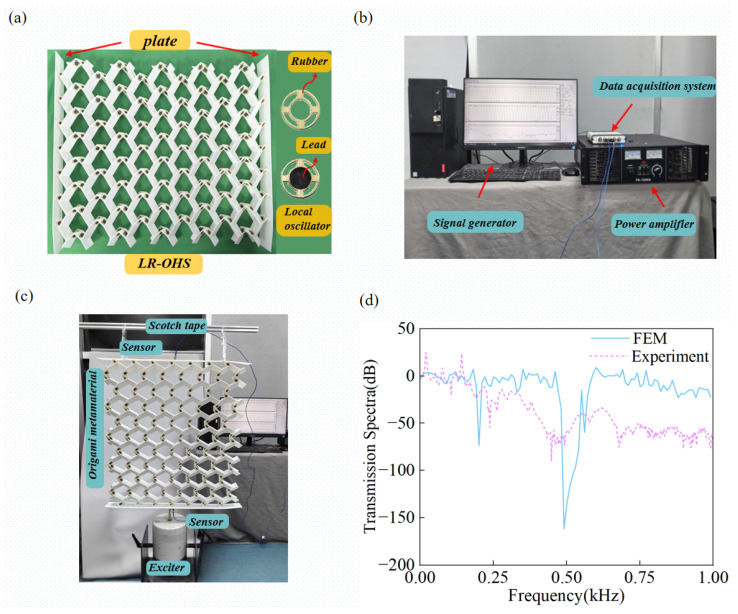
(**a**) Physical picture of LR-OHS; (**b**,**c**) Experimental diagram of LR-OHS; (**d**) Comparison of simulation and experimental results.

To verify the bandgap accuracy and demonstrate prominent experimental phenomena of the proposed LR-OHS, the metamaterial specimens are manufactured using 3D printing technology. The purpose of the experiment is to verify the predicted bandgap and demonstrate the attenuation characteristics of the wave in a truly prepared sample. The selected size is based on the optimized parameters from previous numerical studies. As shown in Figure 11a, the experimental specimens are fabricated with dimensions three times larger than those used in numerical simulations. A wall thickness of 2 mm is maintained for the LR-OHS during experimentation. The main structure of the LR-OHS is fabricated from photosensitive resin, with a density of 1160 $\mathrm{kg}/m^{3}$, Young’s modulus of 2.62 GPa, and Poisson’s ratio of 0.4. The spring material is selected as rubber, which is characterized by a density of 1140 $\mathrm{kg}/m^{3}$, a Young modulus of 6.8 MPa, and a Poisson ratio of 0.47. The local resonators are constructed from lead, which is characterized by a density of 11,350 $\mathrm{kg}/m^{3}$, a Young modulus of 16 GPa, and a Poisson ratio of 0.4. As shown in Figure 11a, the lead blocks are tightly bonded within rubber springs to form local resonators. Subsequently, these resonators are firmly adhered into the voids of the origami structure to assemble the locally resonant metamaterial. A 5 × 5 periodic array is printed, with end plates attached on both sides to facilitate the mounting of actuators for excitation input and sensors for output signal measurement. As shown in Figure 11b, sinusoidal signals are generated by a signal generator, amplified by a power amplifier, and then excited by an actuator. The array structure is secured using Scotch tape. Signals are acquired by sensors at both the excitation point on the lower plate and the response point on the upper plate. The data are then transmitted to a desktop computer via a data acquisition system for processing.

As shown in Figure 11b, sinusoidal signals are generated by a signal generator, amplified by a power amplifier, and then excited by an actuator. Signals are acquired by accelerometers at both the excitation point to a desktop computer via a data acquisition system. In the experimental validation, the results from the 5 × 5 periodic array of LR-OHS are compared between experimental measurements and numerical simulations. As shown in Figure 11d, significant wave attenuation regions around 0.25 kHz and 0.50 kHz are exhibited by both the numerical simulation results (blue solid line) and the experimental results (purple dashed line). Good agreement between the experimental and numerical results is observed, and the excellent wave attenuation capability of the proposed LR-OHS is demonstrated.

The minor frequency shift of the second bandgap is due to deviations between the nominal material properties used in simulations and the actual properties of the 3D printed photosensitive resin and cast rubber. Despite these quantitative differences, the experimental results qualitatively validate the existence of two distinct attenuation regions corresponding to the predicted bandgaps.

### 3.3. Impact Cushioning Performance

**Figure 12:**
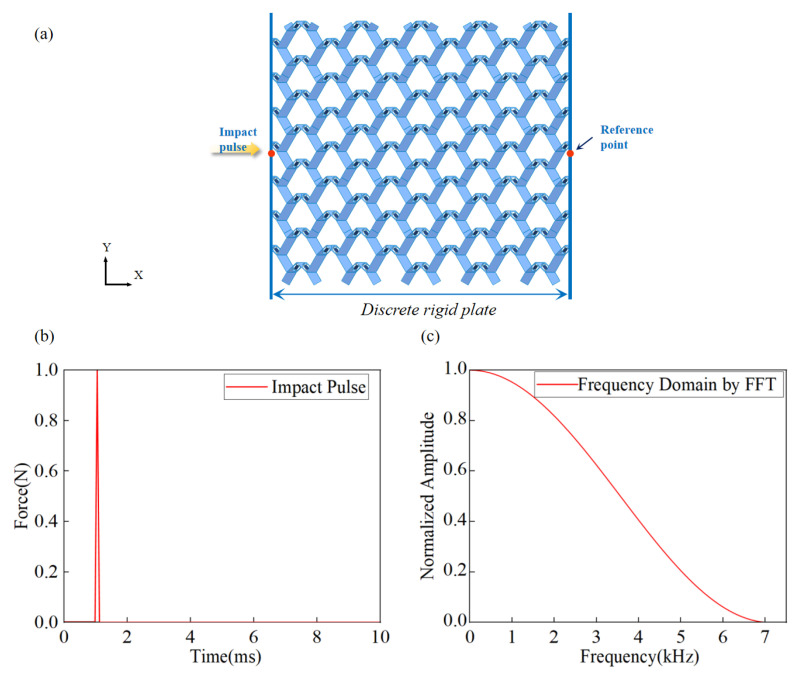
(**a**) Impact pulse loading method and boundary conditions; (**b**) Time domain of impact pulse; (**c**) Frequency domain of impact simulation calculated by Fast Fourier Transform (FFT).

The impact buffering performance of the proposed LR-OHS is investigated in this section. As shown in Figure 12a, a 1000 N impact pulse is applied to the left side of the structure. The response is recorded at a reference point on the right-side rigid plate and converted from the time domain to the frequency domain via FFT, with the results presented in Figure 12b,c. The entire impact event lasted for 70 ms. For clarity of presentation, only the initial 10 ms of the time domain response is shown in Figure 12a. The spectral energy of the impact pulse is primarily distributed within the 0–7 kHz range, which fully encompasses the frequency ranges corresponding to the complete bandgaps RBG1 and RBG2 identified previously.

The following sections present a study on the structure energy dissipation and impact characteristics. The analysis focuses on the effects of the oscillator radius, rubber width, and number of oscillators. As shown in Figure 12a, a metamaterial plate is fabricated by arranging unit cells with varying parameters in an array.

#### 3.3.1. Impact Comparison of Different Models

**Figure 13:**
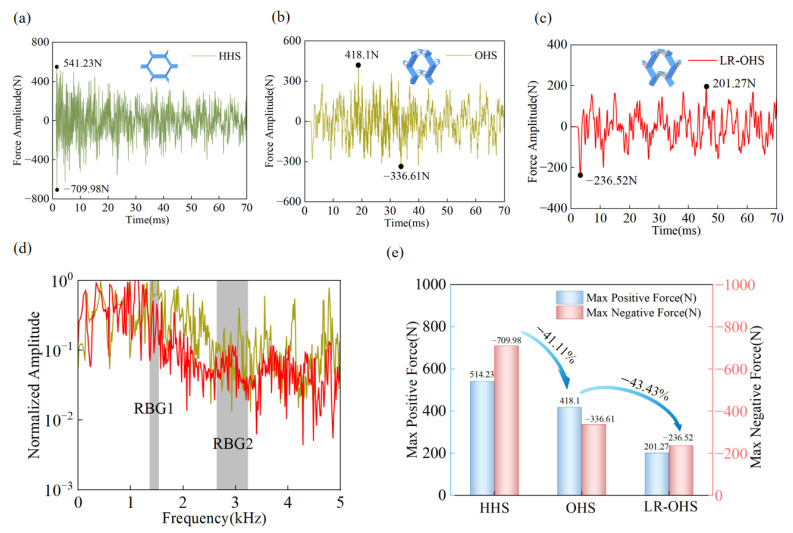
Comparison of reaction forces responses: (**a**) Time domain response of the HHS; (**b**) Time domain response of the OHS; (**c**) Time domain response of the LR-OHS; (**d**) Frequency domain; (**e**) Comparison of the maximum absolute values of reaction forces among the three structures in the simulation.

The impact buffering performance of these structures is analyzed, and the influence of the bandgap on this performance is highlighted. The reaction force curves, measured at an identical reference point on the rigid plate under the same 1000 N impact pulse load, are compared in Figure 13. The reaction force of the HHS in the time domain is represented by the green curve in Figure 13a, the reaction force of the OHS in the time domain is shown by the yellow curve in Figure 13b, and the reaction force of the LR-OHS in the time domain is depicted by the red curve in Figure 13c.

Firstly, as shown in Figure 13e the peak reaction force on the back plate, the values for the HHS, OHS, and LR-OHS are 709.98 N, 418.10 N, and 236.52 N, respectively. This represents a reduction of 41.11% from HHS to OHS and a further reduction of 43.43% from OHS to LR-OHS. Overall, a 66.69% reduction in peak force compared to the HHS is exhibited by the LR-OHS, and a clear and significant decreasing trend across the three structures is demonstrated. Secondly, the time to reach the peak positive reaction force is delayed in the LR-OHS by 43 ms and 26 ms compared to the HHS and OHS, respectively. Thirdly, it is revealed by an analysis of the waveform that upon impact, significantly smaller fluctuations are exhibited by the LR-OHS time domain than those of the HHS and OHS. This waveform smoothing is attributed to energy dissipation. The impact pulse excites local resonances, dissipating kinetic energy through small-amplitude vibrations. Consequently, the peak reaction force transmitted to the back plate is markedly reduced.

To further investigate the effect of the bandgap on impact attenuation and to better highlight the differences between the OHS and LR-OHS structures, the FFT is applied to both to convert their reaction force time domain curves into frequency domain spectra. Figure 13d shows the normalized amplitude of the reaction force versus frequency for both the OHS and LR-OHS. Below approximately 1.40 kHz, the response characteristics of the OHS and the LR-OHS are nearly identical. However, within the frequency range of approximately 1.40 kHz to 1.50 kHz, a pronounced decline is exhibited by the amplitude of the LR-OHS. This frequency range coincides with the RBG1 generated by the structure. Similarly, a significant overall reduction in amplitude is also observed within the RBG2 range. Therefore, it can be concluded that under identical impact pulse loading, impact energy is effectively dissipated and attenuated by the structure with local resonance within its bandgap frequency ranges.

**Figure 14:**
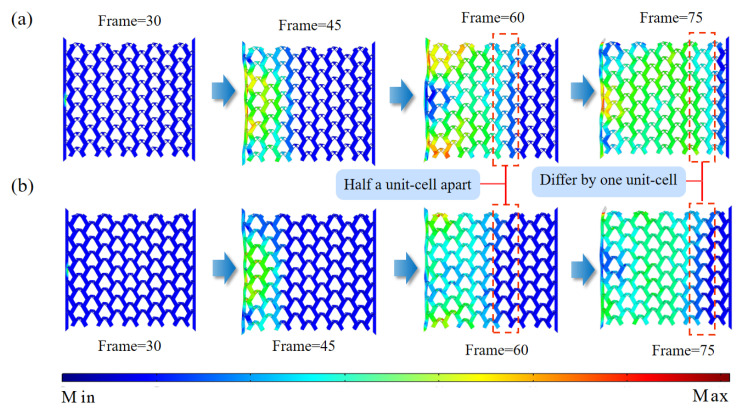
Displacement contour map of the OHS and LR-OHS: (**a**) Displacement fields of the OHS extracted from the finite element software at frame 30, 45, 60, and 75 under pulse impact; (**b**) Displacement fields of the LR-OHS extracted from the finite element software at frame 30, 45, 60, and 75 under pulse impact.

Furthermore, under the identical impact pulse loading, a difference of the shock wave propagation velocity is observed between the OHS and LR-OHS structures. A total duration of 70 ms is possessed by the finite element impact, which is captured over 2000 frames. As shown in Figure 14, the displacement fields of both structures at frames 30, 45, 60, and 75 are presented. Specifically, Figure 14a and Figure 14b show the results for the OHS and LR-OHS, respectively. As the frame number is increased, a disparity in wave propagation velocity between the two structures is made evident. At frame 60, the wavefront in the OHS is nearly half a unit cell ahead of that in the LR-OHS. By frame 75, this lead has been increased to a full unit cell. This phenomenon occurs because, upon entering the LR-OHS, the incident wave energy is not directly transferred to the next unit. Instead, a portion of the energy is dissipated by the local resonators. Consequently, as shown in Figure 14, an additional delay of at least one unit cell time before the impact wave arrives is provided to the object behind the plate by the LR-OHS, thereby resulting in enhanced protection being offered. In summary, the analysis in this section demonstrates that the LR-OHS exhibits exceptional performance in shock absorption and impact mitigation. Valuable insights for future research development and practical applications of origami structures in impact protection are provided by this structure.

#### 3.3.2. The Influence of Important Resonator Radius (r) Parameters on Impact Mitigation

**Figure 15:**
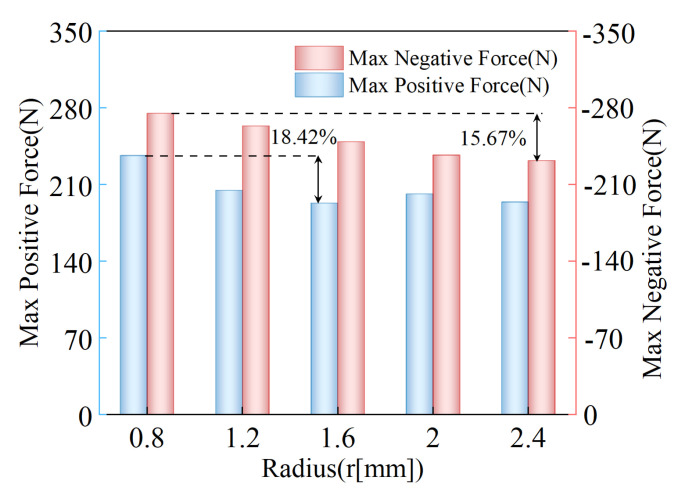
Variation in the peak positive and negative reaction forces with the r.

Figure 15 shows the relationship between the r and the magnitude of the reaction forces. The r is set to five discrete values: 0.8, 1.2, 1.6, 2.0, and 2.4 mm. The peak positive and peak negative reaction forces for each parameter are represented by the blue and red bars, respectively. It is evident that the maximum peak positive reaction force is found at an r of 0.8 mm, while the minimum is found at 1.6 mm. The peak positive force at 1.6 mm is 18.42% lower than that at 0.8 mm. Similarly, the peak negative reaction force is also highest at 0.8 mm and lowest at 2.4 mm. The value at 2.4 mm is 15.76% lower than that at 0.8 mm. It is shown that both the positive and negative peak reaction forces generally decreased with increasing r. This phenomenon occurs because the inertia of the oscillator is increased by a larger r. Essentially, a greater portion of the impact energy is consumed to accelerate the oscillators with higher inertia. The impact energy is effectively dissipated by this process through its conversion into the kinetic energy of the oscillators.

#### 3.3.3. The Influence of Important Resonator Width (b) Parameters on Impact Mitigation

**Figure 16:**
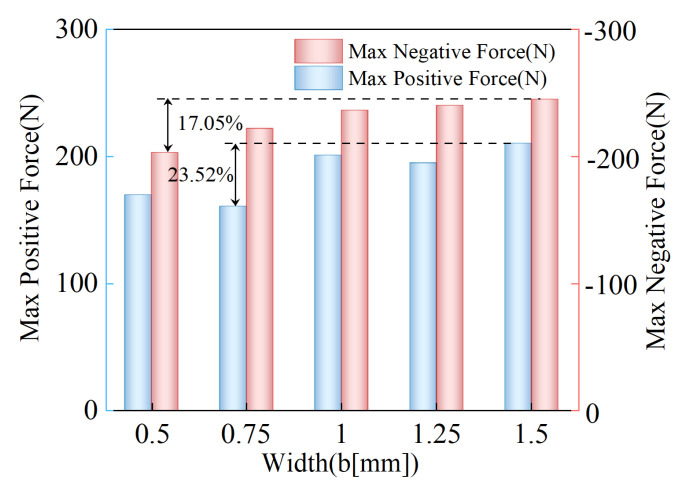
Variation in the peak positive and negative reaction forces with the b.

Figure 16 shows the relationship between the b and the magnitude of the reaction forces. The b is tested at five values: 0.5, 0.75, 1.0, 1.25, and 1.5 mm. The peak positive and peak negative reaction forces for each width are represented by the blue and red bars, respectively. It can be observed that the maximum values for both the positive and negative peak reaction forces are found at a b of 1.5 mm. In contrast, the minimum values are found at 0.75 mm and 0.5 mm for the positive and negative peaks, respectively. The peak positive force at 0.75 mm is 23.52% lower than that at 1.5 mm. Similarly, the peak negative force at 0.5 mm is 17.05% lower than that at 1.5 mm. Furthermore, the peak reaction forces decrease with decreasing b. This trend can be attributed to a reduction in the equivalent spring stiffness as the b decreases. A lower stiffness allows a greater portion of the impact energy to be converted into the kinetic energy of the oscillators and the potential energy stored in the rubber, thereby reducing the reaction forces.

#### 3.3.4. The Influence of Important Resonator Quantities Parameters on Impact Mitigation

**Figure 17:**
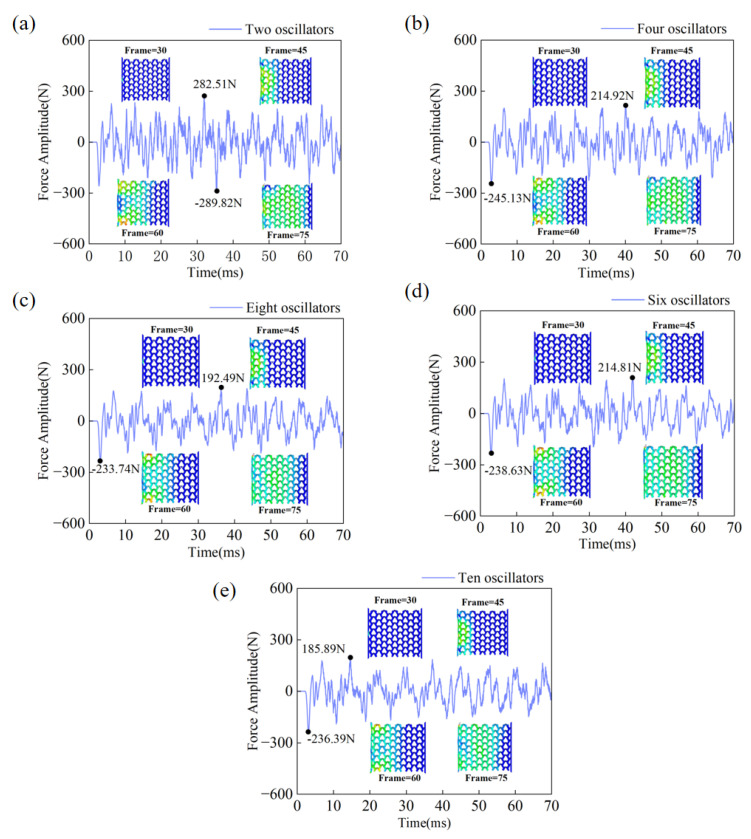
(**a**–**e**) Time domain responses and displacement fields at four selected frames for oscillator counts of 2, 4, 6, 8, and 10, respectively.

**Figure 18:**
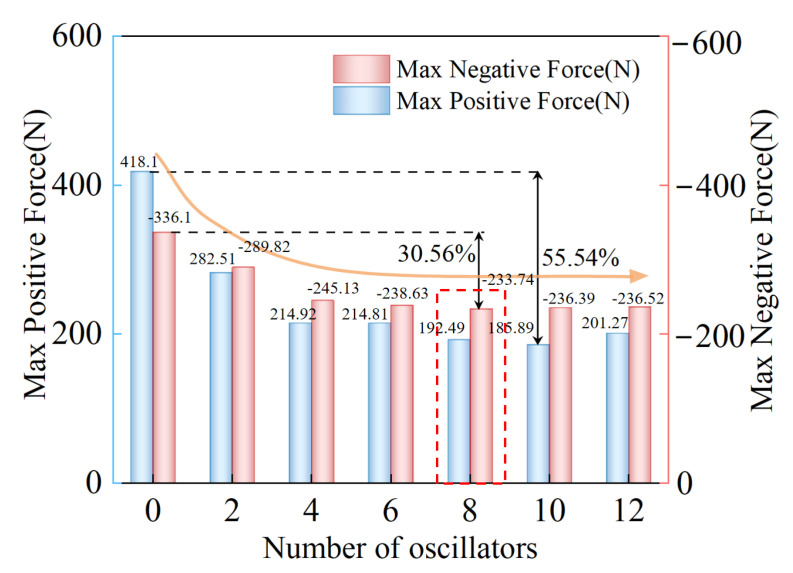
Variation in the peak positive and negative reaction forces with the number of oscillators.

Figure 17a–e show that after introducing the gradient parameter, both the positive and negative reaction forces are significantly reduced. Furthermore, the time domain curves are made considerably smoother. The 70 ms is divided into 2000 frames. The displacement fields for these five gradient configurations are extracted at frames 30, 45, 60, and 75. It is revealed by the analysis of frames 60 and 75 that the wave propagation velocity within the structure is markedly decreased as the number of oscillators is increased. Figure 18 shows that, for a large number of oscillators, the configuration with eight oscillators has the smallest negative reaction force, and the configuration with ten oscillators has the smallest positive reaction force. The peak positive force for the 10-oscillator configuration is 55.54% lower than that of the 0-oscillator baseline. Similarly, the peak negative force for the 8-oscillator configuration is 30.56% lower. Figure 18 shows that the peak reaction forces gradually decrease with an increasing number of oscillators. This decreasing trend is stabilized approximately when the number of oscillators reaches eight, as highlighted by the red dashed box in Figure 18. The cumulative time required to transition each oscillator from rest to an excited state is increased by a greater number of oscillators. Moreover, energy is not transmitted linearly. Instead, it is initially stored and released by the leading oscillators, which subsequently excite the trailing ones. An increasingly complex energy transfer network is created by this process as the number of oscillators is grown. Wave propagation is effectively inhibited and impact resistance is enhanced by the interactions between individual oscillators and the subsequent conversion of energy within them, with both effects being made more pronounced as the number of oscillators is increased. These findings are consistent with the preceding analysis.

## 4. Conclusions

A local-resonant polygonal honeycomb origami metamaterial is presented in this paper, which is developed by introducing the classic Miura-ori pattern to two pairs of edges of the HHS. Through analysis, the first conclusion drawn is that compared to the OHS, the LR-OHS exhibits two complete bandgaps with bandwidths of approximately 95.5 Hz and 0.56 kHz, centered near 1.50 kHz and 3.0 kHz, respectively. Then, two narrow bandgaps merge into a broader one as the resonator radius increases. Notably, the central frequency of the RGB1 shows low sensitivity to variations in radius, whereas that of the RGB2 does not. In the context of impact mitigation, the peak reaction force of the LR-OHS is 43.43% lower than that of the OHS. The arrival time of the positive peak force is also delayed by 26 ms. Moreover, the optimal configuration with eight oscillators is revealed through parameter analysis of the number of oscillators. In summary, the design concepts are extended beyond traditional honeycomb structures by this exploration of origami honeycomb metamaterials. Therefore, this study offers practical design concepts and highly feasible solutions for engineering applications. Future work will focus on experimental validation under actual impact conditions, and further introduce viscoelastic material models and topological optimization methods to realize the collaborative regulation of broadband acoustics and vibration. In addition, the novel metamaterial design based on the origami local resonance mechanism and compression-induced torsion [48] behavior not only exhibits abundant programmable mechanical response properties but also provides new research ideas and implementation approaches for broadband vibration reduction and wave propagation regulation.

## Figures and Tables

**Table 1:** Geometrical parameters.

Geometrical Parameters	Value	Geometrical Parameters	Value
Traditional hexagon side length $L_{2}$ (mm)	14.19	dihedral angle γ (°)	110
Unit cell width $L_{3}$ (mm)	42.57	Folding Angle α (°)	55
Length of the hexagonal side of the unit cell $L_{4}$ (mm)	24.58	Diameter of the round hole 2R (mm)	7
Length of the Miura unit along the x-direction *D* (mm)	14.19	Oscillator diameter 2r (mm)	4
Length of the Miura unit along the y-direction *C* (mm)	14.19	Width of epoxy resin *b* (mm)	1
Side length of a rhombus *A* (mm)	10	Length of epoxy resin $L_{1}$ (mm)	1.5
Angle of the sector β (°)	60	Thickness of the shell *H* (mm)	1

**Table 2:** Material parameters.

Material Parameter	Value	Material Parameter	Value
The density of lead ρ $\left(\mathrm{kg}/m^{3}\right)$	11,350	Poisson’s ratio of rubber μ	0.49
Young’s modulus of lead *E* (pa)	1.6 × 10^10^	Density of epoxy resin ρ $\left(\mathrm{kg}/m^{3}\right)$	1100
Poisson’s ratio of lead μ	0.4	Young’s modulus of epoxy resin *E* (pa)	3 × 10^9^
Density of rubber ρ $\left(\mathrm{kg}/m^{3}\right)$	1000	Poisson’s ratio of epoxy resin μ	0.33
Young’s modulus of rubber *E* (pa)	2.65 × 10^7^		

## Data Availability

The original contributions presented in this study are included in the article. Further inquiries can be directed to the corresponding author.
